# What is different about medical students interested in non-clinical careers?

**DOI:** 10.1186/1472-6920-13-81

**Published:** 2013-06-04

**Authors:** Kyong-Jee Kim, Jae-Hyun Park, Young-Ho Lee, Kyusik Choi

**Affiliations:** 1 Office of Medical Education, Sungkyunkwan University School of Medicine, Irwon-dong Kangnam-gu, Seoul 135-710, Korea; 2 Department of Social and Preventive Medicine, Samsung Biomedical Research Institute, Sungkyunkwan University School of Medicine, 300 Cheoncheon-dong, Jangan-gu, Suwon, Gyeonggi-do 440-746, Korea; 3 Sungkyunkwan University School of Medicine, 300 Cheoncheon-dong, Jangan-gu, Suwon, Gyeonggi-do 440-746, Korea; 4 Department of Social and Preventive Medicine, Sungkyunkwan University School of Medicine, 300 Cheoncheon-dong, Jangan-gu, Suwon, Gyeonggi-do 440-746, Korea

## Abstract

**Background:**

The proportion of medical school graduates who pursue careers other than full-time clinical practice has increased in some countries as the physician’s role has evolved and diversified with the changing landscape of clinical practice and the advancement of biomedicine. Still, past studies of medical students’ career choices have focused on clinical specialties and little is known about their choice of non-clinical careers. The present study examined backgrounds, motivation and perceptions of medical students who intended non-clinical careers.

**Methods:**

A questionnaire was administered to students at six Korean medical schools distributed across all provinces in the nation. The questionnaire comprised 40 items on respondents’ backgrounds, their motivation for and interest in the study of medicine, their perceptions of medical professions, and their career intentions. Data was analyzed using various descriptive and inferential statistics.

**Results:**

In total, 1,388 students returned the questionnaire (60% response rate), 12.3% of whom intended non-clinical careers (i.e., basic sciences, non-clinical medical fields, and non-medical fields). Those who planned non-clinical careers were comparable with their peers in their motivation for studying medicine and in their views of medical professions, but they were less interested in the study of medicine (P < 0.01). The two groups also differed significantly on their perceptions of what was uninteresting about the study of medicine (P < 0.01). The two groups were comparable in gender and entry-level ratios but their distributions across ages and years of study differed significantly (P < 0.01). A majority of respondents agreed with the statements that “it is necessary for medical school graduates to pursue non-clinical careers” and that “medical schools need to offer programs that provide information on such careers.” Still, our finding indicates that medical school curricula do not address such needs sufficiently.

**Conclusions:**

Our study found some differences in backgrounds and perceptions of the study of medicine in medical students interested in non-clinical careers from their peers. Future studies are suggested to enhance our understanding of medical students” choice of non-clinical careers.

## Background

The physician’s role has evolved and diversified as the field of medicine becomes more convergent with other disciplines and the landscape of clinical practice changes [1]. Consequently, the proportion of medical school graduates who pursue careers other than full-time clinical practice has increased in some countries [2] and likely will continue to do so. Therefore, there is a growing need for medical educators to understand factors that influence medical students’ choice of alternative careers in order to better embrace their diverse career interests and needs.

Previous studies have found some internal factors related to medical student’s career choice. Some studies show that student demographics and characteristics are associated with their specialty choice [3-7]. Medical students’ orientations towards career types also influence their specialty choices [8]. In addition to personal attributes, studies have found medical students’ learning experiences such as clerkships and peer tutoring have considerable influence on their career choices [5,9-12]. Furthermore, some studies suggest that external factors such as life style and income influence a student’s choice of medical specialty [13-15].

Although medical students’ career choice has been studied in several countries [16-18], these studies have focused on careers in clinical practice such as internal medicine, surgery, and family practice, and little is known about factors that influence their choice of non-clinical careers (i.e., careers other than full-time clinical practice). Therefore, there is a strong need to study medical students interested in non-clinical careers for a better understanding of their career planning needs. To that end, the present study examined backgrounds, motivation and perceptions of medical students who intended non-clinical careers. In more detail, the research questions for the present study are:

• Are there differences in background between students who intend clinical careers and those who intend non-clinical careers?

• Do these groups differ in their motivation for and interest in studying medicine?

• Do these groups differ in their perceptions of medical professions?

• Do these groups differ in their attitudes towards career plans?

## Methods

### Setting and participants

A cross-sectional study was carried out on medical students in South Korea. There are 41 medical schools around the nation in South Korea with over 14,000 registered students. Both undergraduate-entry and graduate-entry tracks exist in the current Korean medical education system. Graduate-entry students go through four years of study in medical school, whereas two-year premed courses are preceded for undergraduate-entry students. As of 2013, about a third of all Korean medical schools offer undergraduate-entry medical programs, another one-third have graduate-entry programs, and the others have programs with both tracks.

We selected six medical schools across the nation for the sample of the present study. One is a graduate-entry program, another one is an undergraduate-entry program, and the others have both tracks. Two of these schools are located in the metropolitan areas and the others are in midsize cities; they were selected from provinces across the nation to obtain representative geographical differences. In terms of differences in the curriculum across these medical schools, one has a fully problem-based curriculum for its pre-clinical courses in Year 2 and the others have hybrid problem-based curricula, in which problem-based learning is integrated into somewhat traditional curriculum.

### The research instrument

A survey instrument was used to examine the influence of medical students’ backgrounds, motivation and perceptions in their career intentions. A literature search was conducted of existing research instruments suited for the present study in both English and Korean. No research instrument was available in English, so we decided to adopt one in Korean [19] for the present study. This questionnaire was developed by Park in the 1990s for a national survey of medical students on their perceptions of medical education and medical practice. This original questionnaire comprised 49 items, and we used most of the items in this questionnaire for comparison of results. Nine items regarding respondents’ perceptions of diseases, health, and the healthcare system were left out of the questionnaire for the present study because they were beyond the scope of this study.

We conducted a pilot study of the questionnaire to check for the clarity of the items. Seven medical students took part in this pilot study and no unclear or unfeasible items were found. Consequently, the questionnaire for the present study was composed of 40 items with five subscales. The questionnaire for the present study is translated into English by the authors and is provided in Additional file 1. Ten items regarding respondent demographics, which include names of Korean medical schools and provinces, are excluded from this English version.

The first part of the questionnaire (items 1-4) asked about respondent’s motivation for getting into medical school. These items related to when the respondent decided to go to medical school and what influenced his or her decision. The second part of the questionnaire (items 5-9) asked respondent’s level of interest in studying medicine, what was interesting and uninteresting about it, and his or her intention to switch to other majors. In the third part of the questionnaire (items 10-25), respondents were asked to indicate their career intentions, reasons behind such choices and their attitudes towards career planning. In the fourth part of the questionnaire (items 26-30), participants were asked about their perceptions of medical professions, including merits and demerits of being a doctor and the social status of doctors. The last part of the questionnaire (items 31-40) consisted of questions regarding participant demographics and backgrounds.

### Data collection and analysis

The questionnaire was administered in October and November, 2010. The questionnaire was distributed to all students in the medical schools under study, except for one medical school in which third and fourth year students could not participate due to time constraints. The questionnaire was self-administered by the students and was answered anonymously. The questionnaires, which included consent forms, were distributed to each medical school and were collected a week later. Students who agreed to participate signed the consent form, completed the survey and submitted it in a sealed envelope. The present study was approved by the institutional review board of Samsung Seoul Hospital (2010-11-001).

Data were analyzed using SPSS for Windows (SPSS, Inc., Chicago, IL, USA). Both descriptive and inferential statistics were conducted to analyze the data. In particular, the chi-square analysis and Student’s *t*-test were performed to investigate differences in participant backgrounds and perceptions between the groups who intended clinical careers and those who intended non-clinical careers. All significance was tested at the 95% level of confidence.

## Results

### Survey respondents and their career intentions

A total of 1,388 students completed and returned the questionnaire (60% response rate). The respondents varied in their years of study and their ages ranged from under 20 to over 30. Of these respondents, 33% (*n* = 460) were female and 67% (*n* = 927) were male. Eighteen percent of the respondents (*n* = 253) were graduate-entry students; the others (82%) were undergraduate-entry students.

Respondents were asked to choose one out of four options for their career intentions; basic science, clinical science, non-clinical medical professions, and non-medical professions. Table 1 illustrates the distribution of respondents’ career intentions. 87.7% (*n* = 1,217) of the respondents answered that they intended careers in clinical practice (i.e., the clinical careers group). The most frequently selected clinical specialties were internal medicine (*n* = 352), psychiatry (*n* = 111), and orthopedic surgery (*n* = 101). Meanwhile, 12.3% of those surveyed (*n* = 171) answered they intended careers outside clinical practice (i.e., the non-clinical careers group). Those who selected non-clinical careers were categorized into three sub-groups; (1) 42 chose basic sciences (e.g., physiology, biochemistry, and pharmacology); (2) 90 chose non-clinical medical professions (e.g., healthcare administration, medical engineering, and medical education); and (3) 39 chose careers in non-medical fields (e.g., business administration, law, and finance).

**Table 1:** Respondent’s future career intentions

**Clinical careers**		**Non-clinical careers**			**Total**
**Primary care**	**Specialty**	**Basic sciences**	**Non-clinical medical fields**	**Non-medical fields**	
34 (2.4%)	1,183 (85.3%)	42 (3.0%)	90 (6.5%)	39 (2.8%)	1,388 (100%)
1,217 (87.7%)		171 (12.3%)			

Fifty-five percent (*n* = 94) of those in the non-clinical careers group indicated their personal interest in the given fields as the main reason for intending such careers. The second most frequent answer varied according to the areas of work that they chose: 31% (*n* = 13) of those who intended careers in basic sciences answered they were interested in research; 20% (*n* = 18) of those who intended careers in non-clinical medical fields indicated that they liked exploring new areas; 22% (*n* = 8) of those who intended non-medical fields indicated that they had not intended clinical practice as their full-time job.

Table 2 shows the results of cross-tabulation between respondents’ backgrounds and their career intentions. The gender ratios were comparable (*χ*^2^ = .002, *P* = .51) and the ratios of respondents in terms of their entry levels (i.e., undergraduate-entry vs. graduate-entry) were also comparable (*χ*^2^ = 32.8, *P* = .46) between the clinical careers group and the non-clinical careers group. Yet, the two groups differed significantly in their distributions across ages (*χ*^2^ = 32.8, *P* < .001) and across years of study (*χ*^2^ = 18.9, *P* = .002).

**Table 2:** Respondent’s backgrounds and their career intentions

	**Future career intentions**		**Total**	***P *value**
	**Clinical practice**	**Non-clinical careers**		
Gender				.512
Female	403 (29.1%)	57 (4.1%)	460 (33.2%)	
Male	813 (58.6%)	114 (8.2%)	757 (66.8%)	
Age (years)				< .001
20 or younger	161 (12.0%)	40 (3.0%)	201 (15.0%)	
21-25	759 (56.5%)	87 (6.5%)	846 (63.0%)	
26-30	213 (15.9%)	30 (2.2%)	243 (18.1%)	
31 or older	48 (3.6%)	5 (0.4%)	53 (3.9%)	
Entry level				.458
Undergraduate	990 (71.4%)	143 (10.3%)	1,133 (81.7%)	
Graduate	226 (16.3%)	27 (1.9%)	253 (18.3%)	
Year of study				.002
Premed 1	207 (14.9%)	50 (3.6%)	257 (18.5%)	
Premed 2	242 (17.4%)	34 (2.4%)	276 (19.9%)	
Year 1	268 (19.3%)	34 (2.4%)	302 (21.8%)	
Year 2	239 (17.2%)	32 (2.3%)	271 (19.5%)	
Year 3	158 (11.4%)	14 (1.0%)	172 (12.4%)	
Year 4	103 (7.4%)	7 (0.5%)	110 (7.9%)	
Total	1,217 (87.7%)	171 (12.3%)	1,388 (100%)	

### Respondents’ motivation for and interest in the study of medicine

Respondents indicated what motivated them to get into medical school by choosing an answer from a variety of options, including interest in learning biomedicine, job security, advice from family and friends, providing care for humans, inspired by doctors. The most frequently selected answer was job security (22.4%), followed by interest in learning biomedicine (18.7%). We found no significant difference in respondents’ motivation for studying medicine between the clinical careers group and the non-clinical careers group (*χ*^2^ = 26.7, *P* = .85).

Participants rated their levels of interest in the study of medicine on a five-point scale from 1 = “very uninterested” to 5 = “very interested.” Thirteen percent (*n* = 182) of the respondents answered that they were not interested in the study of medicine, whereas 62% (*n* = 861) found it very or somewhat interesting. The two groups differed significantly in their levels of interest in studying medicine, where *M* = 3.68 (SD = .96) for the clinical careers group and *M* = 3.39 (SD = 1.14) for the non-clinical careers group (*t* = 3.1, *P* < .01).

Respondents were asked to indicate what was interesting about studying medicine by choosing an answer from a variety of options. The most frequently selected answer was learning about the human body (50%), followed by acquiring knowledge relevant to daily lives (21%), patient contact (14%), and acquiring specialized knowledge (12%). Respondent perceptions of what was interesting about studying medicine were not significantly different between the groups (*χ*^2^ = 3.71, *P* = .45).

Table 3 illustrates respondent perceptions of what was uninteresting about studying medicine. The most frequently selected answer was there was too much to learn (28%), followed by too much to memorize (27%), lack of diverse perspectives in medical courses (18%), and lack of personal life (16%). The two groups differed significantly in their perceptions of what was uninteresting about the study of medicine (*χ*^2^ = 19.1, *P* < .01). In particular, a higher portion of those in the non-clinical careers group answered there was too much to memorize in studying medicine than their peers (25.7% vs. 32.7%).

**Table 3:** Responses on what was uninteresting about the study of medicine

**Response options**	**Future career intentions**		**Total**
	**Clinical fields**	**Non-clinical fields**	
Too much to learn	357 (25.7%)	37 (2.7%)	394 (28.4%)
Too much to memorize	313 (22.6%)	56 (4.0%)	369 (26.6%)
Lack of diverse perspectives	212 (15.3%)	37 (2.6%)	249 (17.9%)
Lack of personal life	208 (15.0%)	14 (1.0%)	222 (16.0%)
Rigid assessments	91 (6.6%)	18 (1.3%)	109 (7.9%)
Lack of educational facilities	20 (1.4%)	5 (0.4%)	25 (1.8%)
Others	16 (1.1%)	4 (0.3%)	20 (1.4%)
Total	1,217 (87.7%)	171 (12.3%)	1,388 (100.0%)

Ten percent of the total respondents (*n* = 135) indicated that they would have considered switching their major had they been given the opportunity. Those who intended non-clinical careers were more likely to have thought about switching their major than their peers (8.1% vs. 21.1%, where *χ*^2^ = 28.5, *P* < .001). In particular, 24% (*n* = 10) of those with career intentions in basic sciences, 13% (*n* = 12) of those with career intentions in non-clinical medical fields, and 36% (*n* = 14) of those with career intentions in non-medical fields indicated that they would consider switching major. The ratios of respondents having considered switching major differed significantly among these three groups (*χ*^2^ = 8.59, *P* = < .05).

### Respondent perceptions of medical professions

Respondents were asked to indicate their opinions of the merit and demerit of being a doctor to investigate their perceptions of medical professions. Respondents stated that the most significant merits of being a doctor were the humanitarian nature of the work (27%), prestige (20%), autonomy of work (18%), and job security (17%). Meanwhile, respondents chose lack of personal life (52%), job stress (33%), negative public perceptions of doctors (7%), and low income levels (7%) as the most significant demerits of being a doctor. We found no statistically significant differences in the respondent’s perceptions of merits and demerits of being a doctor between the groups, where *χ*^2^ = 8.12, *P* = .32 and *χ*^2^ = 4.10, *P* = .39, respectively.

Respondents rated their views of the social status of doctors in terms of prestige, social influence, power, and income on a 10-point scale (1 = “very low,” 10 = “very high”). The means were 7.15 for prestige (SD = 1.70), 5.80 for social influence (SD = 1.87), 5.34 for power (SD = 1.78), and 7.16 for income (SD = 1.44). We found no significant differences in the respondent’s perceptions of the social status of doctors between the groups in all four aspects of social status mentioned above, where *P* values ranged from .93 to .09. Respondents also rated their perceptions of the ideal social status of doctors on the same scale. Respondents’ views of the actual social status of doctors were significantly lower than those of their perceptions of the ideal one, where *p* < .001 in all four aspects of social status. These gaps in the respondents’ views of actual and ideal social status of doctors were comparable between the groups, where *P* values ranged from .331 to .932.

### Respondents’ attitudes towards career plans

A vast majority of the total respondents (88%) indicated that they had given thought to their future careers either sometimes or frequently, which were rated on a three-point scale with 1 being rarely and 3 being frequently. Those who intended non-clinical careers thought about their career plans more frequently than their peers who intended clinical careers (*χ*^2^ = 26.9, *P* < .001). Additionally, 83.6% of those surveyed answered that it was necessary for medical schools to offer programs that help them explore non-clinical careers.

76% of the total respondents agreed with the statement that it would be necessary for medical school graduates to pursue non-clinical careers; in particular, those who intended non-clinical careers agreed more strongly with this statement than their peers (*χ*^2^ = 11.6, *P* < .01). 37% of the total respondents indicated that they would likely succeed in their careers if they chose non-clinical professions, whereas 53% said they were unsure of it, and 10% indicated unlikely. Those who intended non-clinical careers were more certain of their success in such careers than their peers (*χ*^2^ = 6.21, *P* < .05).

Those who answered that they intended non-clinical careers (*n* = 39) were asked how they were preparing for careers in such areas. The most frequently selected answer was reading books (*n* = 21), followed by participating in school clubs (*n* = 8) or in other social activities (*n* = 7). Only three respondents answered that they were doing so by taking courses in medical school. Additionally, the most frequently selected answer on the difficulties that respondents encountered when preparing for non-clinical careers was lack of time to pursue interests in such areas while studying medicine (*n* = 22), followed by others’ expectations for them to pursue clinical careers (*n* = 9), and lack of information on non-clinical careers (*n* = 5).

## Discussion and conclusions

The present study shows that the percentage of Korean medical students who intend non-clinical careers has increased over the past decade; from 7.3% in Park’s study [19] to 12.3%. This finding is similar to other studies of US medical students which show the national trend of an increase in medical students intending non-traditional careers [1,2]. Still, we found a trend of gradually decreasing number of students intending non-clinical careers over the years of study in medical school. It may be that students become more interested in clinical practice as they gain more clinical experience over the course of study. As medical students’ views of clinical specialties change over time [20,21], the same may be true of their views of non-clinical careers. This trend is also consistent with our finding that younger students are more interested in non-clinical careers than older students. Yet, our study indicates other demographic backgrounds such as gender and entry-level are not strong predictors of medical students’ preferences for non-clinical careers.

We found no significant differences in students’ views of medical professions between those who intended non-clinical careers and their peers, but there were some differences in their perceptions of the study of medicine. First, students with intentions in non-clinical careers are less interested in the study of medicine than their peers, although the two groups are comparable in their motivation for getting into medical school. Second, those who intend non-clinical careers differ from their peers in their perceptions of what is uninteresting about the study of medicine. In particular, a higher portion of the students who intended non-clinical careers found there was too much to memorize in studying medicine than their peers. One possible interpretation for these findings is the influence of medical students’ cognitive and learning styles in their learning and career choices. The literature indicates that students’ interest in learning is related to their cognitive and learning styles, which also influence their learning outcomes [22]. Additionally, research suggests that medical students’ cognitive and learning styles influence their specialty choices [22,23]. Yet, these studies are limited to clinical careers and little is known about the influence of medical students’ cognitive and learning styles on their preference for non-clinical careers. Future research is warranted to understand the association between an individual’s cognitive and learning style and his or her intention in non-clinical careers.

Our findings indicate that a majority of medical students think it is important for them to consider careers outside clinical practice and that medical schools need to offer programs that provide information on such careers. Still, our finding indicates that medical school curricula do not address such needs sufficiently. Therefore, it is suggested that academic and career counseling for medical students be more tailored to individual needs based on an understanding of their personal interests.

Limitations of the present study should be acknowledged. First, the sample size was limited in that only six out of 41 medical schools across the nation were included. Yet, we believe that we minimized selection bias by selecting a sample across the provinces in the nation and by trying to minimize disparity in the response rate of each medical school under investigation. Second, this study reflects the perspectives of Korean medical students, which may be affected by the health care environment that they are in. To help compare the healthcare environment of Korea with that of others, it can be argued that the quality of health care in Korea is on par with that of developed countries. Additionally, there have been growing concerns regarding reduced compensation for doctors due to changes in the health care system and a rapidly growing number of medical school graduates over the past decade. This also has caused increased competitions to obtain residency in some preferred medical specialties.

Despite the limitations of the present study, it has shed light on backgrounds and perceptions of medical students who intend non-clinical careers. Still, the literature also indicates a possible link between the student’s personality type and his or her career choice [7]. Thus, future study of factors that influence medical students’ intentions in non-clinical careers that include their personality traits may help enhance our understanding of medical students’ choice of non-clinical careers.

## Competing interests

All authors read and approved the final manuscript.

## Authors’ contributions

KK collaborated on the analysis and interpretation of data and drafted the manuscript. JP contributed to conception, design, acquisition, and interpretation of data. YL contributed to acquisition, analysis, and interpretation of data. KY contributed to design and data acquisition. All authors critically reviewed the manuscript for revision and approved the version to be published.

## Pre-publication history

The pre-publication history for this paper can be accessed here:

http://www.biomedcentral.com/1472-6920/13/81/prepub

## Supplementary Material

Additional file 1 Questionnaire on Medical Students’ Career Intentions. Click here for file
