# Supplementary material for: What is different about medical students interested in non-clinical careers?
Source: BMC Med Educ. 2013 Jun 4;13:81. doi: 10.1186/1472-6920-13-81 (PMC3679731; doi:10.1186/1472-6920-13-81)
Supplement: Additional file 1 — Questionnaire on Medical Students’ Career Intentions. [file 1472-6920-13-81-S1.docx]

Appendix 1. Questionnaire on Medical Students’ Career Intentions

**Questionnaire on Medical Students’ Career Intentions**

**I. Motivation for Getting into Medical School**

1. When did you decide to go to medical school?

O Before entering elementary school O In elementary school

O In junior high school O In high school O After graduating from high school

O In college O After graduating from college

2. Did anyone influence your decision to go to medical school?

O Yes O No

3. If you answered “Yes” to the question above, who influenced your decision?

O Father O Mother O Teacher

O Relative O Friend O Other ( )

4. What motivated you the most to go to medical school?

O It fit my interests

O I got a good score in Korea SAT or had a good high school GPA

O Because of the job security of doctors

O Advice from family and friends

O Providing care for humans

O Inspired by doctors

O Others _______________

**II. Interest in the Study of Medicine**

5. How would you rate your level of interest in the study of medicine?

O Very interested O Somewhat interested O Neutral

O Uninterested O Very uninterested

6. Should you have a chance to choose what to study in college again, what would you choose?

O Humanities or social science O Pharmacy O Business

O Medicine O Law O Oriental medicine O Science

O Dentistry O Engineering O Arts or athletics O Others

7. Would you like to change your major now, if you are allowed to?

O Yes O No

8. Choose what you feel the most interesting and uninteresting about the study of medicine.

| The most interesting thing | The most uninteresting thing |
| --- | --- |
| O Learning about the human body  O Acquiring specialized knowledge  O Patient contact  O Acquiring knowledge relevant to daily lives  O Others | O Too much to learn  O Too much to memorize  O Lack of diverse perspectives  O Lack of personal life  O Rigid assessments  O Lack of educational facilities  O Others |

9. Do you think it is necessary for medical school to offer education (seminars or lectures) on disciplines outside medicine for students?

O Yes O No

**III. Questions Regarding Career Intentions**

10. How often have you thought about your future careers?

O Rarely O Sometimes O Frequently

11. Should you choose a career from the following areas at this point in time, what would it be?

O Basic science O Clinical science

O Non-clinical medical fields (e.g., medical education, medical engineering, healthcare administration) O Non-medical fields

◆ If you answered “Basic science” to the question #11, please answer the following questions 12 & 13.

12. What discipline in basic sciences would you like to choose?

O Anatomy O Physiology O Biochemistry

O Microbiology, including immunology O Parasitology

O Pathology O Pharmacology O Preventive medicine

13. What motivated you the most to choose a career in basic science?

O Fits my interest and aptitude
O Higher chance to become a medical school faculty

O Social prestige

O Interested in research

O Not confident about seeing patients

O Others _______________

◆ If you answered “Clinical science” to the question #11, please answer the following questions #14 & 15.

14. If you would choose a career in clinical medicine, which of the following would you choose?

O Primary care medicine O A specialty area

15. If you would choose a medical specialty, what would it be? (Choose two from the following areas in order of your preference)

| Specialty area | 1^st^ choice | 2^nd^ choice |
| --- | --- | --- |
| Internal medicine  Pediatrics  Dermatology  General surgery  Thoracic and cardiovascular surgery  Neurological surgery  Neurology  Psychiatry  Orthopedic surgery  Plastic surgery  Obstetrics & gynecology  Ophthalmology  Urology  Family medicine  Rehabilitative medicine  Orhinolaryngology  Anesthesiology  Clinical pathology  Emergency medicine  Nuclear medicine  Industry medicine  Radiology  Preventive medicine  Laboratory medicine | O  O  O  O  O  O  O  O  O  O  O  O  O  O  O  O  O  O  O  O  O  O  O  O | O  O  O  O  O  O  O  O  O  O  O  O  O  O  O  O  O  O  O  O  O  O  O  O |

16. What motivated you the most to choose the area of clinical medicine above?

O Fits my interest O High income levels

O Good quality of life O A low risk of malpractice

O Helping a lot of patients O Social prestige

O Others _______________

◆ If you answered “Non-clinical medical fields” to the question #11, please answer the following questions #17 & 18.

17. If you would choose a non-clinical medical career, what would it be?

O Medical education O Healthcare administration

O Medical engineering O Medical economics

O History of medicine O Forensic medicine O Medical ethics

O Others _______________

18. What motivated you the most to choose the non-clinical medical career?

O Fits my interest O Not confident about seeing patients

O Like exploring new areas O Job stability O Social prestige

O Others ______________

◆ If you answered “Non-medical fields” to the question #11, please answer the following questions #19 - 22.

19. If you would choose a career in a non-medical field, what would it be?

O Government or public organizations O Lawyer O Media or entertainment O Research O Trade or commerce O Service areas O Finance

O Social welfare O Healthcare industry O Educator

O Agriculture, forestry, or fishery O Business administration or accounting

O Arts and culture O Others ______________

20. What motivated you the most to choose a career in non-medical fields?

O Fits my interest O Low income levels of doctors

O Unsatisfactory work environment for doctors

O Unfavorable public perceptions of doctors

O Had not intended clinical practice as a full-time job

O Others ______________

21. How are you preparing for careers in non-clinical areas?

O Participate in school clubs

O Take lessons about the area of my interest outside the school

O Take or took college courses in disciplines outside of medicine

O Read books for self-study

O Work part-time or take part in social activities in the area of my interest

O Others ______________

22. What was the most difficult thing that you encountered in preparing for non-clinical careers?

O Others’ expectations for them to pursue clinical practice

O Lack of information on non-clinical careers

O Lack of time to pursue interests in non-clinical fields while studying medicine

O Others ______________

23. Which type of workplace do you intend to work for the most?

O Medical school/academic health center O Private practice

O General hospital O Public healthcare center

O Public health administration O Research institute

O International missionary O Others ______________

24. Do you think it is necessary for medical school graduates to pursue non-clinical careers?

O Yes O No O Neutral

25. How likely are you to succeed in your career should you choose one outside clinical practice?

O Very likely O Unsure O Very unlikely

**IV. Attitudes towards Medical Professions**

26. Which of the followings do you think are merits of doctors? (Choose two from the following in order of your preference)

| Options | 1^st^ choice | 2^nd^ choice |
| --- | --- | --- |
| High social status  High income  Social power  The humanitarian nature of work  Autonomy of work  Job stability  Others ( ) | O  O  O  O  O  O  O | O  O  O  O  O  O  O |

27. In your opinion, which of the followings do medical students think are merits of doctors? (Choose two from the following in order of your preference)

| Options | 1^st^ choice | 2^nd^ choice |
| --- | --- | --- |
| High social status  High income  Social power  The humanitarian nature of work  Autonomy of work  Job stability  Others ( ) | O  O  O  O  O  O  O | O  O  O  O  O  O  O |

28. What do you think is the actual social status of doctors in Korea?

|  | Very Low Very High |
| --- | --- |
| Prestige | ① ② ③ ④ ⑤ ⑥ ⑦ ⑧ ⑨ ⑩ |
| Social influence | ① ② ③ ④ ⑤ ⑥ ⑦ ⑧ ⑨ ⑩ |
| Power | ① ② ③ ④ ⑤ ⑥ ⑦ ⑧ ⑨ ⑩ |
| Income | ① ② ③ ④ ⑤ ⑥ ⑦ ⑧ ⑨ ⑩ |

29. What do you think is the ideal social status of doctors in Korea?

|  | Very Low Very High |
| --- | --- |
| Prestige | ① ② ③ ④ ⑤ ⑥ ⑦ ⑧ ⑨ ⑩ |
| Social influence | ① ② ③ ④ ⑤ ⑥ ⑦ ⑧ ⑨ ⑩ |
| Power | ① ② ③ ④ ⑤ ⑥ ⑦ ⑧ ⑨ ⑩ |
| Income | ① ② ③ ④ ⑤ ⑥ ⑦ ⑧ ⑨ ⑩ |

30. What do you think is the most significant demerit of doctors?

O Lack of personal life

O Job stress

O Negative public perceptions of doctors

O Low income levels

O Others ( )
